# Brown fat depots in adult humans remain static in their locations on PET/CT despite changes in seasonality

**DOI:** 10.14814/phy2.13284

**Published:** 2017-06-06

**Authors:** Terence A. Jones, Narendra L. Reddy, Sarah C. Wayte, Oludolapo Adesanya, Georgios K. Dimitriadis, Charles E. Hutchinson, Thomas M. Barber

**Affiliations:** ^1^ Clinical Sciences Research LaboratoriesWarwick Medical SchoolUniversity of WarwickCoventryCV2 2DXUnited Kingdom; ^2^ Department of RadiologyUniversity Hospitals Coventry and WarwickshireCoventryCV2 2DXUnited Kingdom; ^3^ Department of Medical PhysicsUniversity Hospitals Coventry and WarwickshireCoventryCV2 2DXUnited Kingdom

**Keywords:** Brown adipose tissue, colocalization, human, positron‐emission tomography

## Abstract

Active brown adipose tissue (BAT) in humans has been demonstrated through use of positron emission tomography with 2‐deoxy‐2‐(fluorine‐18) fluoro‐D‐glucose integrated with computed tomography (^18^F‐FDG PET/CT) scans. The aim of our study was to determine whether active human BAT depots shown on ^18^F‐FDG PET/CT scans remain static in their location over time. This was a retrospective study. Adult human subjects (*n* = 15) who had had ^18^F‐FDG PET/CT imaging (*n* = 38 scans in total) for clinical reasons were included on the basis of ^18^F‐FDG uptake patterns consistent with BAT activity. For each subject, ^18^F‐FDG BAT uptake pattern on serial ^18^F‐FDG PET/CT images was compared to an index ^18^F‐FDG PET/CT image with the largest demonstrable BAT volume. Object‐based colocalization was expressed as Mander's correlation coefficient (where 1 = 100% overlap, 0 = no overlap). Distribution of ^18^F‐FDG BAT activity over time and across multiple ^18^F‐FDG BAT scans was equivalent in 60% (*n* = 9) of the subjects. The degree of consistency in the pattern of ^18^F‐FDG BAT uptake in each subject over time was greater than expected by chance in 87% (*n* = 13) of the subjects (pair‐wise agreement 75–100%, Fleiss’ *κ* 0.4–1). The degree of BAT colocalization on serial scans was greater than that expected by chance in 93% (*n* = 14) of the subjects (mean Mander's coefficient 0.81 ± 0.21 [95% CI]). To our knowledge, our study provides the most conclusive evidence to date to support the notion that active BAT depots in humans (volumes and activities of which were measured through use of ^18^F‐FDG PET/CT scans) remain static in location over sustained periods.

## Introduction

Brown adipose tissue (BAT) is both anatomically and functionally distinct from white adipose tissue (WAT) (Cannon and Nedergaard 2004; Ronti et al. 2006; Saely et al. 2012). Through generating heat from non‐shivering thermogenesis (Del Mar Gonzalez‐Barroso et al. 2000; Enerback 2010), BAT has therapeutic potential to facilitate weight loss, and may persist into adulthood in a large proportion of humans. Previously, our own group published the first proof of concept study in a living human adult to demonstrate the utility of magnetic resonance imaging (MRI) in the discernment of BAT from adjacent WAT based on differences in water:fat ratios between the two tissues (Reddy et al. 2014). This MRI‐based anatomical approach to BAT imaging contrasts with the more widely published imaging modality of positron emission tomography with 2‐deoxy‐2‐(fluorine‐18) fluoro‐D‐glucose integrated with computed tomography (^18^F‐FDG PET/CT), which only demonstrates *active* BAT (rather than BAT anatomy) through its uptake of ^18^F‐FDG. Such reported studies have demonstrated that BAT activity in human adults predominates within certain anatomical locations that include cervical, thoracic and paravertebral regions (Barrington and Maisey 1996). Comparison of images from ^18^F‐FDG PET/CT scans from the same subject have demonstrated the facultative nature of human BAT, with BAT depots becoming active within minutes of stimulation through cold exposure (van Marken Lichtenbelt et al. 2009).

Although human BAT activity as a whole is clearly inducible and facultative in response to stimuli such as cold exposure (Cannon & Nedergaard 2004), an important unanswered question relates to the *pattern* of BAT activity in each individual over time: it is not clear whether the distribution of BAT depot activity in each individual human adult (with demonstrable BAT activity) remains fixed and static in location over time, or is susceptible to changeability and impermanence. Such understanding and insight into patterns of BAT activity are relevant for future therapeutic approaches to manipulate BAT. Factors that would perhaps predispose to a changeable pattern of BAT depot activity over time may include trans‐differentiation of WAT into new beige fat depots (separate from established BAT depots), or perhaps other hitherto unknown local paracrine factors that influence activity of individual BAT depots. These hypotheses however are purely speculative, and we accept that transdifferentiation (of WAT surrounding established BAT depots for example) may also be consistent with a pattern of BAT depot activity that is fixed and static over time. The aim of our study was to determine whether active human BAT depots shown on ^18^F‐FDG PET/CT scans remain static in their location over time.

## Methods

### Subject selection

Subjects were selected for inclusion in this study retrospectively on the basis of identified ^18^F‐FDG uptake within presumed BAT depots (indicative of active BAT) on images from all ^18^F‐FDG PET/CT scans (*n = *3317) performed for clinical reasons (mainly oncology assessments) between June 2007 and August 2012, in patients attending University Hospitals Coventry and Warwickshire (UHCW). For this purpose, a keyword search was employed using the terms ‘brown adipose tissue’ and ‘brown fat’ to identify relevant images from their associated radiology reports written by a Consultant Radiologist with specific expertise in nuclear imaging. It is usual practice at UHCW for the presence of active BAT to be reported within radiology reports of ^18^F‐FDG PET/CT images. A total of 175 ^18^F‐FDG PET/CT scans from 152 patients were identified to demonstrate ^18^F‐FDG uptake within presumed active BAT depots. Of these 152 patients, 15 had evidence of ^18^F‐FDG uptake within presumed active BAT depots on multiple scans (*n = *38 scans in total) performed at different times. Clinical details of these 15 subjects are outlined in Table 1. All investigations were conducted in accordance with the guidelines in the Declaration of Helsinki. The study was approved by a local Research Ethics Committee in the United Kingdom.

**Table 1:** Demographics of patients with multiple ^18^F‐FDG BAT positive PET/CT scans (adapted with permission of OmniScriptum GmbH (Jones 2016))

Patient	Age at first PET/CT (years)	Sex	BMI (kg/m^2^) at index PET/CT	Serum glucose (mmol/L) at index PET/CT	Diagnosis
A	57	Male	26.1	5.6	Bronchogenic cancer
B	14	Male	19.1	5.1	Hodgkin's lymphoma
C	20	Female	35.6	4.8	Hodgkin's lymphoma
D	52	Female	26.2	6.0	Malignant melanoma
E	29	Female	23.3	4.8	Hodgkin's lymphoma
F	20	Male	23.8	4.6	Hodgkin's lymphoma
G	85	Female	26.1	5.6	Bronchogenic cancer
H	58	Male	21.4	6.0	Gastro‐intestinal stromal tumour
I	22	Female	25.5	4.4	Hodgkin's lymphoma
J	17	Female	21.6	4.8	Hodgkin's lymphoma
K	37	Female	24.1	5.3	Breast cancer
L	43	Female	26.6	5.1	Oesophageal cancer
M	25	Female	25.3	8.9	Hodgkin's lymphoma
N	36	Female	21.0	4.8	Cervical cancer
O	75	Female	23.0	5.1	Bowel cancer

### ^18^F‐FDG PET/CT image acquisition

^18^F‐FDG PET/CT scanning was performed on a combined GE Discovery STE PET/CT scanner (General Electric Medical Systems, Milwaukee). In accordance with standard administration and acquisition protocol, patients were fasted for 6 h prior to scanning. ^18^F‐FDG was administered intravenously one hour prior to scan acquisition (mean injected dose 362 ± 33 MBq; range 103–505 MBq). Static emission data were obtained from the skull base to mid thigh level (with the arms elevated where possible) with unenhanced spiral CT scans at 3.3 mm slice thicknesses for attenuation correction.

### ^18^F‐FDG PET/CT image analysis

On analyzing images from ^18^F‐FDG PET/CT scans, the reporting Consultant Radiologist reported presence of presumed active BAT when there was avid ^18^F‐FDG uptake at a location that was greater than background level (Standard Uptake Value [SUV] >1.0 g/mL), within an anatomical region that was consistent with the presence of active BAT (confirmed WAT on CT, with attenuation of between −100 and −10 Hounsfield units). Images from ^18^F‐FDG PET/CT scans (*n* = 175) that had reports of avid ^18^F‐FDG uptake within presumed active BAT depots were then reviewed by a second radiologist (co‐author TAJ) using a GE ADW Advantage 4.3 Workstation (GE Healthcare, Milwaukee) to determine if ^18^F‐FDG uptake characteristic of active BAT occurred within each of four anatomical compartments as described by Ouellet et al. (2011): neck/supraclavicular fossae (SCF); mediastinum; paravertebral, and; peri‐renal regions.

Within each of the ^18^F‐FDG PET/CT images from the 15 subjects who had ^18^F‐FDG uptake within presumed active BAT depots on multiple scans (*n* = 38) over time, ^18^F‐FDG BAT volumes were calculated using Mirada XD 3.4 (Mirada Medical Ltd, Oxford, United Kingdom). Regions of interest (ROIs) corresponding to ^18^F‐FDG BAT deposits were selected semi‐automatically by defining iso‐contours around putative BAT depots, with a threshold SUV of 2.5 g/mL to minimize artefactual ‘bleeding’ of FDG activity into adjacent tissues, using a similar technique to that described by van Marken Lichtenbelt et al. (2009) and Huang et al. (2011). For each of the 15 subjects who had ^18^F‐FDG active BAT uptake on multiple scans, the ^18^F‐FDG PET/CT image with the largest volume of ^18^F‐FDG BAT uptake was identified as the ‘index’ scan, which formed a benchmark against which other ‘subordinate’ scans taken at other times from the same subject were compared.

### Statistical analysis

For each of the 15 patients with 18F‐FDG BAT uptake on multiple 18F‐FDG PET/CT scans (*n* = 38 scans), the pattern of BAT activity on serial scans over time was compared using pair‐wise percent agreement and Fleiss’ kappa using the online calculator ‘Reliability Calculator for 3 or more coders’ (ReCal3) (Freelon, 2010) (http://dfreelon.org/utils/recalfront/recal3/). This technique provided an assessment of the degree to which BAT activity pattern remains constant and static over time. Subordinate 18F‐FDG PET/CT images were registered to their respective index 18F‐FDG PET/CT image using a combination of automatic rigid and non‐rigid registration as appropriate using Mirada XD 3.4, and verified visually using an inbuilt visualization tool. Quantitative object‐based colocalization analysis (Lachmanovich *et al*., 2003) (a well‐established technique used in fluorescence microscopy to demonstrate that particular proteins are associated with certain organelles) was employed in our study to evaluate colocalization of individual 18F‐FDG active BAT depots on these registered serial 18F‐FDG PET/CT images from each individual over time, using ImageJ 1.45 (U.S. National Institutes of Health, Bethesda, Maryland, USA) (Rasband, 1997–2004) ‘Just Another Colocalization Plugin’ (JACoP) 2.0 (Bolte & Cordelières, 2006). The degree of fractional overlap between segmented 18F‐FDG active BAT depots on subordinate scans with respect to those depots on the index scan was calculated, and expressed as a ‘Mander's colocalization coefficient’ (where 1 = 100% overlap and 0 = no overlap).

To determine whether the degree of colocalization of 18F‐FDG active BAT depots across serial scans for each individual was greater than would be expected by chance, the calculated ‘Mander's colocalization coefficients’ were compared with those derived from randomized images using the Confined Displacement Algorithm (CDA) plugin (Ramirez *et al*., 2010) for Image J. Probability distribution curves were generated against which the baseline colocalization coefficients were compared. Calculated colocalization coefficients were considered significant if they were >95% of the coefficients generated from randomized images.

## Results

### Anatomical patterns and distribution of ^18^F‐FDG active BAT

Amongst the ^18^F‐FDG PET/CT images (*n* = 175) showing presumed BAT activity, ^18^F‐FDG uptake within active BAT occurred most commonly within cervical/SCF regions (162/175 [93%] of scans) followed by the paravertebral region (143/175 [82%] of scans). The timing of the serial ^18^F‐FDG PET/CT scans (*n* = 38) for the 15 subjects with multiple images showing ^18^F‐FDG active BAT, including the anatomical locations of active BAT are shown in Table 2. The time intervals between ^18^F‐FDG PET/CT scans for each of these 15 subjects ranged between 3 months and 4 years (mean time interval between first and last scan was 16 months). In 14 out of these 15 subjects, the index scan showed ^18^F‐FDG BAT uptake within at least as many anatomical compartments as the subordinate scans. Of the 15 subjects with multiple ^18^F‐FDG BAT positive scans, 9 subjects (60%) showed ^18^F‐FDG BAT uptake within the same anatomical compartments on serial scans (100% pair‐wise agreement, Fleiss’ *κ* = 1), which is much greater than what would be expected by chance (data shown in Tables 2 and 3). The most notable exemplar was subject F, who showed 100% concordance in ^18^F‐FDG active BAT distribution across 4 separate ^18^F‐FDG PET/CT scans performed over a 13‐month period. Subject B also showed a very high level of concordance across 5 scans (mean pair‐wise agreement 90%, Fleiss’ *κ* = 0.76). Subjects I, J and O showed comparatively lower levels of inter‐rater agreement, although discordance was limited to a single anatomical compartment resulting in fair‐to‐moderate agreement, according to the criteria of Landis and Koch (1977). There was poor concordance in the pattern of BAT activity for 2 subjects (H and L) resulting in poor agreement (Fleiss’ *κ* = −0.14 and −0.33 respectively).

**Table 2:** Anatomical distribution patterns of a ^8^F‐FDG BAT uptake across four anatomical compartments on serial PET/CT scans (adapted with permission of OmniScriptum GmbH (Jones 2016))

Patient	Scan date	SUVmax	^18^F‐FDG BAT volume (mL)	Anatomical compartment^b^			
				1	2	3	4
A	January 2009	6.2	31.8^a^	+^c^	+	+	−
	November 2011	8.2	30.8	+	+	+	−
B	March 2009	8.2	70.7	+	+	+	−
	September 2009	11.1	94.3^a^	+	+	+	−
	November 2009	12.7	72.9	+	+	+	−
	January 2010	6.3	24.0	+	+	−	−
	December 2010	4.0	18.6	+	+	+	−
C	November 2009	3.9	2.0	+	+	−	−
	December 2009	7.4	49.4^a^	+	+	−	−
D	November 2010	16.1	217.4	+	+	+	−
	March 2011	10.3	324.8^a^	+	+	+	−
E	September 2010	5.9	47.9^a^	+	+	−	−
	March 2011	4.6	9.1	+	+	−	−
F	January 2011	13.4	84.0^a^	+	+	+	−
	June 2011	12.5	33.9	+	+	+	−
	September 2011	10.2	44.3	+	+	+	−
	February 2012	4.4	32.7	+	+	+	−
G	April 2012	4.7	20.1^1^	−	+	−	+
	October 2012	3.3	2.5	−	+	−	+
H	April 2008	8.1	43.6^a^	+	+	−	+
	May 2011	5.0	43.5	+	+	+	+
I	February 2011	8.1	35.8	+	+	+	−
	August 2011	22.2	258.9^a^	+	+	+	+
	March 2012	5.1	29.5	+	+	+	−
J	February 2011	14.8	409.7^a^	+	+	+	+
	April 2011	4.6	25.8	+	+	+	−
	July 2011	6.2	53.8	+	+	+	−
K	November 2011	8.4	73.3^a^	+	+	+	−
	February 2012	4.7	10.9	+	−	+	−
L	February 2008	10.4	111.1^1^	+	+	+	+
	February 2012	4.5	20.0	+	−	+	−
M	March 2009	6.3	8.0	+	−	−	−
	June 2010	10.5	37.0^a^	+	−	−	−
	February 2009	4.5	1.7	+	−	−	−
N	March 2009	4.6	23.2	+	+	+	+
	August 2009	5.8	61.7^a^	+	+	+	+
O	September 2011	2.9	0.9	−	+	+	−
	May 2009	5.9	18.9^a^	+	+	+	−

a Index scan.

b 1, cervical/supraclavicular; 2, paravertebral, 3, mediastinal; 4, peri‐renal compartments.

c ‘+’denotes presence of ^18^F‐FDG BAT in that compartment, and ‘−’ denotes absence of ^18^F‐FDG BAT.

**Table 3:** Inter‐rater reliability calculations for serial ^18^F‐FDG BAT positive PET/CT scans (reprinted with permission of OmniScriptum GmbH (Jones 2016))

Patient	Mean pairwise agreement (%)	Fleiss’ *κ*	Fleiss (expected agreement)	Fleiss (observed agreement)
A	100	1.00	0.63	1.00
B	90	0.76	0.58	0.90
C	100	1.00	0.50	1.00
D	100	1.00	0.63	1.00
E	100	1.00	0.50	1.00
F	100	1.00	0.63	1.00
G	100	1.00	0.50	1.00
H	75	−0.14	0.78	0.75
I	83	0.40	0.72	0.83
J	83	0.40	0.72	0.83
K	100	1.00	0.63	1.00
L	50	−0.33	0.63	0.50
M	100	1.00	0.63	1.00
N	100	Undefined due to invariant values		
O	75	0.47	0.53	0.75

### Colocalization analysis

**Figure 1:**
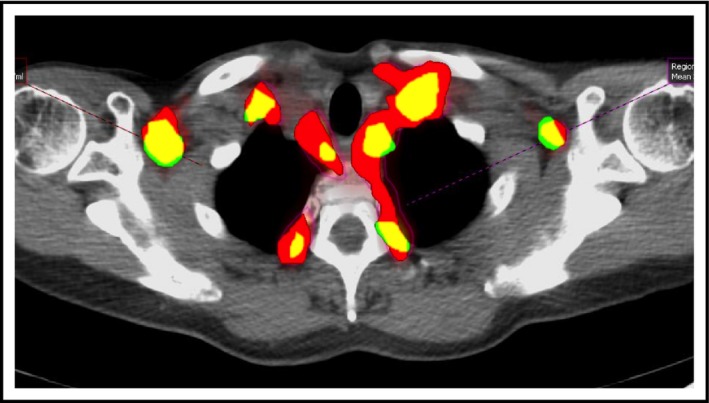
Axial PET/CT of the upper thorax for patient B showing superimposed segmented ^18^F‐FDG BAT regions of interest from the index (red) and subordinate PET/CT scans (green), with areas of colocalization shown as yellow (reprinted with permission of OmniScriptum GmbH (Jones 2016)).

**Figure 2:**
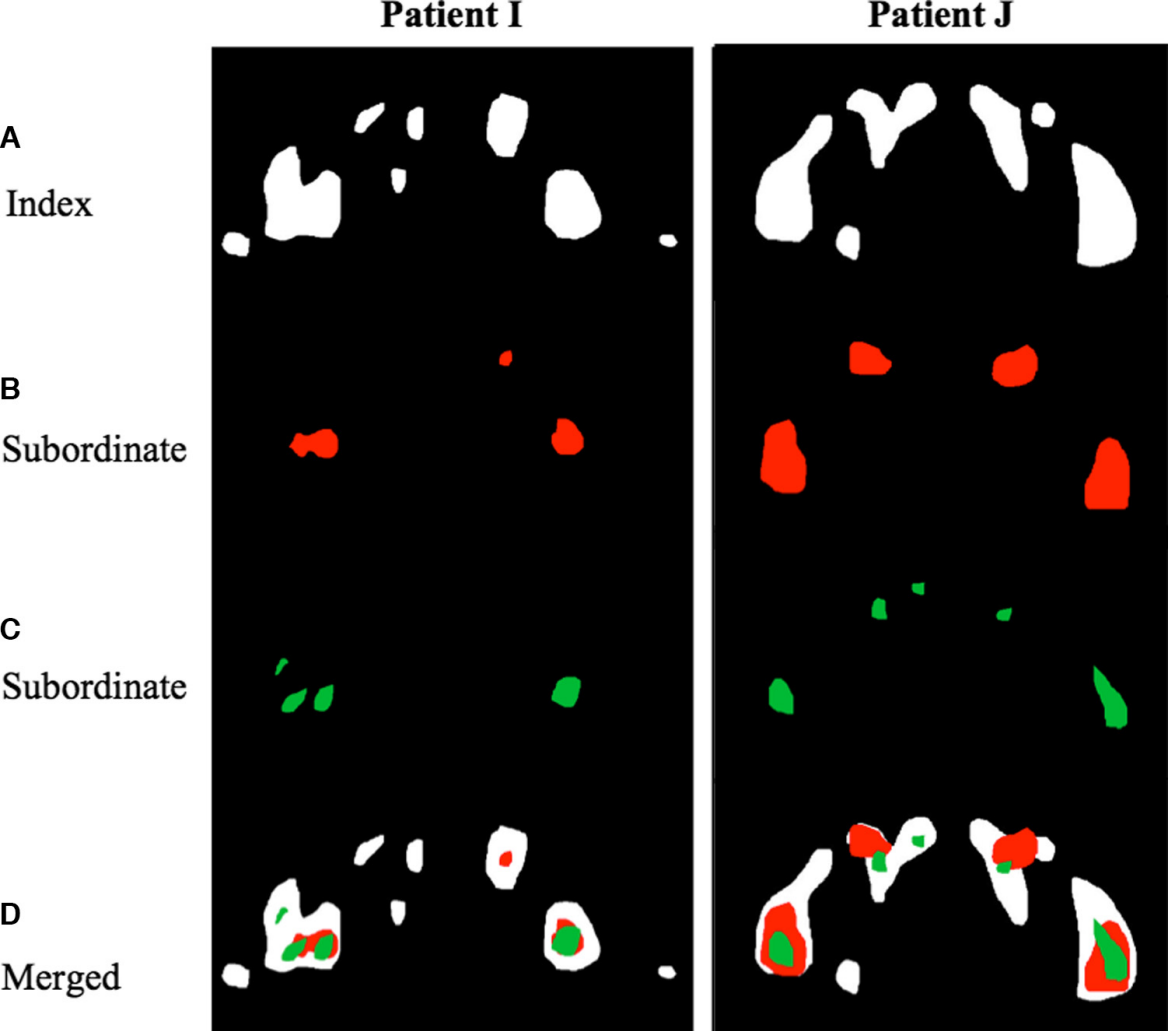
Serial PET/CT scans for two exemplar patients (I and J) showing segmented ^18^F‐FDG BAT from respective index scans (A), subordinate scans (B and C), with merged segmented images showing a high degree of visual colocalization (D).

A typical colocalized image of the upper thorax for subject B is shown in Figure 1, with areas of colocalization between the ^18^F‐FDG BAT ROIs on the index scan and subordinate scan shown as yellow (non‐colocalizing ROIs on the subordinate and index scans are shown as green and red respectively). To determine colocalized ROIs for each subject, ROIs from subordinate scans were compared with those from the index scan. In 14 subjects (93%), the majority of ROIs on subordinate scans colocalized with ROIs on the index scans. Those subjects with a high degree of colocalization who had multiple subordinate scans (subjects B, F, I, J and M) tended to show consistently high levels of colocalization across all their subordinate scans. This is exemplified by two subjects illustrated in Figure 2: subjects I and J who each had 3 scans performed over a 13‐month and 5‐month period respectively. In both cases ^18^F‐FDG BAT ROIs on subordinate scans tended to fall within the larger ^18^F‐FDG BAT ROIs on the index scan.

**Figure 3:**
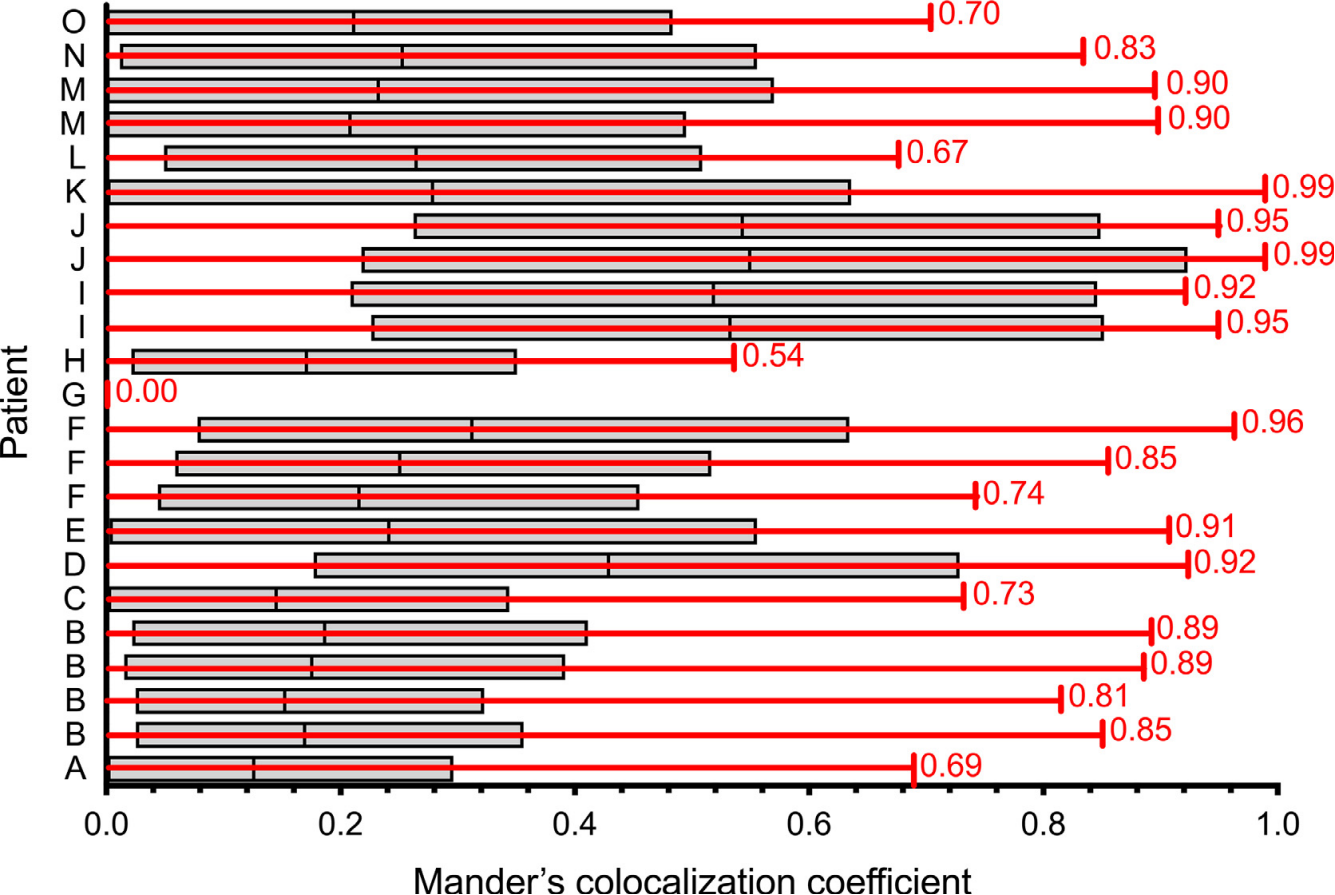
Mander's colocalization coefficients for each patient's subordinate scan with respect to the index scan (red), with colocalization coefficients derived from randomized images (grey boxes denoting 95% confidence intervals) showing colocalization to be higher than chance for 22/23 subordinate scans (adapted with permission of OmniScriptum GmbH (Jones 2016)).

Mean Mander's colocalization coefficient was 0.81 ± 0.21. With one exception (patient G), quantitative colocalization analysis yielded Mander's colocalization coefficients greater than what would be expected by chance (Figure** 3). There were 19/23 (83%) subordinate scans in 11 subjects that yielded coefficients ≥0.7 (meaning that ≥70% of the ^18^F‐FDG BAT voxels on subordinate scans in those subjects coincided with ^18^F‐FDG BAT on the respective index scan). In 2 of the remaining subjects, Mander's colocalization coefficient was high at 0.69 and 0.67.

## Discussion

To our knowledge, our study provides the most conclusive evidence to date to support the notion that active BAT depots in human adults (volumes and activities of which were measured through use of ^18^F‐FDG PET/CT scans) remain static and constant in location over long periods of time, often years. Although variable between subjects, anatomical distribution patterns of ^18^F‐FDG BAT activity within each subject also remained static and constant over time.

Data from serial ^18^F‐FDG PET/CT scans were reported by Rousseau et al. (2006). In this study, patients with breast cancer (*n* = 33) were imaged, and ^18^F‐FDG PET/CT scans (*n* = 5) performed for each patient. Although ^18^F‐FDG BAT uptake was shown to be unrelated to age or outdoor temperature, ^18^F‐FDG BAT uptake was identical on all 5 ^18^F‐FDG PET/CT scans in only a small minority (15%, *n* = 5) of the patients included in this study (Rousseau et al. 2006). In a prospective study design to assess the effects of cold exposure and capsinoid ingestion over a 6‐week period on human BAT activity, energy expenditure and body fat mass, Yoneshiro and colleagues showed a negative correlation between BAT activity and body fat mass (Yoneshiro et al. 2011). Although prospective in design, this study had a much shorter duration than ours, and assessment of changes in active BAT location over time was hampered by some of the scans showing BAT inactivity (due to lack of stimulation) (Yoneshiro et al. 2011). Finally, Ouellet and colleagues demonstrated in a group of 328 ^18^F‐FDG BAT positive human subjects that outdoor temperature, age, sex, BMI and diabetes status influences ^18^F‐FDG BAT activity (Ouellet et al. 2011). However, the authors did not report on colocalization data for BAT depots from serial scans on each subject (Ouellet et al. 2011).

The ^18^F‐FDG PET/CT scan when used for clinical purposes is an inherently limited imaging modality for detecting ^18^F‐FDG uptake within active BAT depots. Therefore the detection of active BAT in our retrospective study was entirely opportunistic. The likelihood of detecting metabolically active BAT on PET/CT varies with age, gender, body mass index and time of day, while activity (SUVmax) varies with environmental temperature and age (Jones 2016). We sought to address this inherent limitation (which undermines the reliability of ^18^F‐FDG PET/CT as an effective imaging reference standard for active BAT in humans) through use of an ‘index’ scan. For each subject, the ‘index’ scan provided a reasonably accurate indication of the anatomical pattern of ^18^F‐FDG BAT uptake on subordinate scans. We acknowledge that, due to its retrospective design, scans were performed during all seasons and therefore with variable environmental temperatures, and that this represents a limitation of our study. However, it seems unlikely that seasonal variations in environmental temperature between scans for each subject would have changed our main conclusion that active BAT depots remain static and constant in their locations over time. Indeed, our observation that metabolically active BAT depots remained static in their locations over long periods of time despite variations in both environmental parameters and patient demographics strengthens our conclusions.

Although our sample size of 15 patients (38 PET/CT scans) may be considered small, to identify metabolically active BAT on serial PET/CT scans is unusual. An exhaustive search of 3317 PET/CT scans identified 15 patients in whom BAT was evident on serial PET/CT scans.

A further potential limitation of our study is that analysis of ^18^F‐FDG PET/CT images does not facilitate perfect colocalization. Acquisition of images from PET and CT (the 2 components of ^18^F‐FDG PET/CT) are metachronous, with durations of >30 min and a few seconds respectively. There is therefore potential for some ^18^F‐FDG BAT activity on each scan to be mis‐registered and appear erroneously outside fat regions due to subtle differences in patient positioning during each scan. Image segmentation on the basis of CT attenuation could then exclude any mis‐registered ^18^F‐FDG BAT uptake. Furthermore, despite standard operating procedures, it is reasonable to expect small differences in patient positioning between each scan, which may not be fully remedied by *post hoc* image registration. This is an unlikely source of bias, however, as both index and subordinate scans in each subject would be affected similarly by such small variations in patient positioning.

In segmenting ^18^F‐FDG BAT, we used a higher SUV cut‐off than other authors (Ouellet et al. 2011) to maximize specificity, which has the potential to introduce a type II error by underestimating the true extent of BAT activity. The point prevalence of ^18^F‐FDG BAT in our sample was 5.3% (Jones et al. 2016) which is comparable to those reported in other retrospective PET‐based studies (Au‐Yong et al. 2009; Cypess et al. 2009; Ouellet et al. 2011; Pace et al. 2011; Cronin et al. 2012; Mei 2012), which in turn are consistently lower than in dedicated prospective studies (van Marken Lichtenbelt et al. 2009; Virtanen et al. 2009). Therefore, it seems likely that even our index scans underestimated the true extent and volume of ^18^F‐FDG BAT depots. However, this probable underestimation of the true extent of active BAT on each scan is unlikely to have changed our main conclusion, given that a standard SUV was employed for each scan, and the standard SUV threshold employed throughout our study would not be expected to change the observed distribution of active BAT between scans for each subject.

An inherent problem with the ^18^F‐FDG PET/CT imaging modality for detecting active BAT in humans is that enhanced uptake of ^18^F‐FDG is non‐specific for active BAT and appears in other metabolically active tissues. To address this ^18^F‐FDG uptake was only considered as active BAT if it occurred within WAT in anatomically well‐defined regions for human active BAT as described by Ouellet et al. (2011). ^18^F‐FDG uptake that appeared in other regions was disregarded for the purposes of our study. Although histological and immunohistochemical (Uncoupling Protein‐1, [UCP1] staining) confirmation of BAT was not possible in our study given its retrospective design, we have previously provided such confirmation in a human adult subject using the ^18^F‐FDG PET/CT imaging modality through examination of excised tissue specimens from a region of increased ^18^F‐FDG uptake, anatomically corresponding to active BAT (Reddy et al. 2014).

In summary, we provide evidence that not only does the distribution of metabolically active BAT in adult humans remain fairly static over sustained periods of time, but individual BAT deposits colocalize to a high degree, despite changes in seasonality.

## Conflict of Interest

All authors have nothing to declare, and there is no duality of interest.
